# Markers of metabolic health and gut microbiome diversity: findings from two population-based cohort studies

**DOI:** 10.1007/s00125-021-05464-w

**Published:** 2021-06-10

**Authors:** Semi Zouiouich, Erikka Loftfield, Inge Huybrechts, Vivian Viallon, Panayiotis Louca, Emily Vogtmann, Philippa M. Wells, Claire J. Steves, Karl-Heinz Herzig, Cristina Menni, Marjo-Riitta Jarvelin, Rashmi Sinha, Marc J. Gunter

**Affiliations:** 1 Section of Nutrition and Metabolism, International Agency for Research on Cancer-WHO, Lyon, France; 2 Metabolic Epidemiology Branch, Division of Cancer Epidemiology and Genetics, National Cancer Institute, Rockville, MD USA; 3 Department of Twin Research, King’s College London, London, UK; 4 Research Unit of Biomedicine, Medical Research Center (MRC), University of Oulu, University Hospital, Oulu, Finland; 5 Department of Gastroenterology and Metabolism, Poznan University of Medical Sciences, Poznan, Poland; 6 Department of Epidemiology and Biostatistics, MRC-PHE Centre for Environment and Health, School of Public Health, Imperial College London, London, UK; 7 Center for Life Course Health Research, Faculty of Medicine, University of Oulu, Oulu, Finland; 8 Unit of Primary Health Care, Oulu University Hospital, OYS, Oulu, Finland; 9 Department of Life Sciences, College of Health and Life Sciences, Brunel University London, London, UK

**Keywords:** Faecal microbiome, HOMA-IR, Insulin resistance, Metabolic health

## Abstract

**Aims/hypothesis:**

The gut microbiome is hypothesised to be related to insulin resistance and other metabolic variables. However, data from population-based studies are limited. We investigated associations between serologic measures of metabolic health and the gut microbiome in the Northern Finland Birth Cohort 1966 (NFBC1966) and the TwinsUK cohort.

**Methods:**

Among 506 individuals from the NFBC1966 with available faecal microbiome (16S rRNA gene sequence) data, we estimated associations between gut microbiome diversity metrics and serologic levels of HOMA for insulin resistance (HOMA-IR), HbA_1c_ and C-reactive protein (CRP) using multivariable linear regression models adjusted for sex, smoking status and BMI. Associations between gut microbiome diversity measures and HOMA-IR and CRP were replicated in 1140 adult participants from TwinsUK, with available faecal microbiome (16S rRNA gene sequence) data. For both cohorts, we used general linear models with a quasi-Poisson distribution and Microbiome Regression-based Kernel Association Test (MiRKAT) to estimate associations of metabolic variables with alpha- and beta diversity metrics, respectively, and generalised additive models for location scale and shape (GAMLSS) fitted with the zero-inflated beta distribution to identify taxa associated with the metabolic markers.

**Results:**

In NFBC1966, alpha diversity was lower in individuals with higher HOMA-IR with a mean of 74.4 (95% CI 70.7, 78.3) amplicon sequence variants (ASVs) for the first quartile of HOMA-IR and 66.6 (95% CI 62.9, 70.4) for the fourth quartile of HOMA-IR. Alpha diversity was also lower with higher HbA_1c_ (number of ASVs and Shannon’s diversity, *p* < 0.001 and *p* = 0.003, respectively) and higher CRP (number of ASVs, *p* = 0.025), even after adjustment for BMI and other potential confounders. In TwinsUK, alpha diversity measures were also lower among participants with higher measures of HOMA-IR and CRP. When considering beta diversity measures, we found that microbial community profiles were associated with HOMA-IR in NFBC1966 and TwinsUK, using multivariate MiRKAT models, with binomial deviance dissimilarity *p* values of <0.001. In GAMLSS models, the relative abundances of individual genera *Prevotella* and *Blautia* were associated with HOMA-IR in both cohorts.

**Conclusions/interpretation:**

Overall, higher levels of HOMA-IR, CRP and HbA_1c_ were associated with lower microbiome diversity in both the NFBC1966 and TwinsUK cohorts, even after adjustment for BMI and other variables. These results from two distinct population-based cohorts provide evidence for an association between metabolic variables and gut microbial diversity. Further experimental and mechanistic insights are now needed to provide understanding of the potential causal mechanisms that may link the gut microbiota with metabolic health.

**Graphical abstract:**

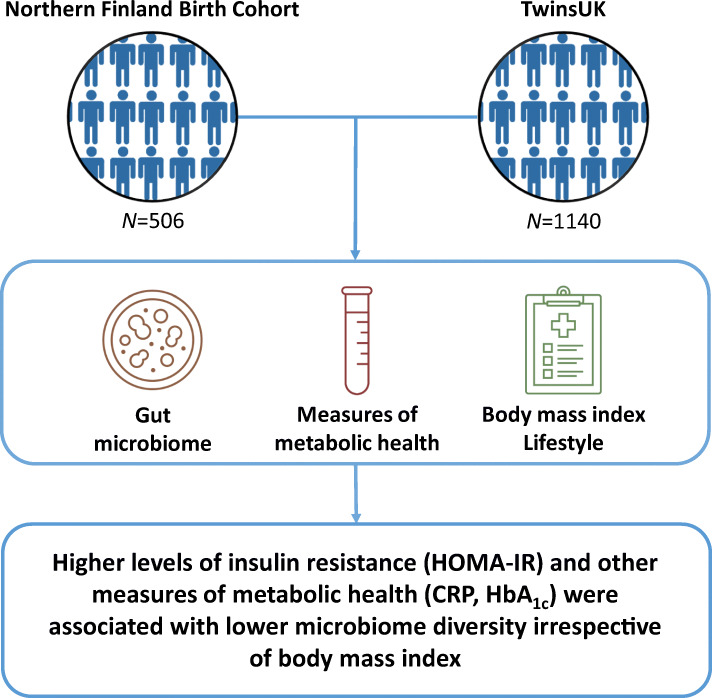

**Supplementary Information:**

The online version contains peer-reviewed but unedited supplementary material available at 10.1007/s00125-021-05464-w.

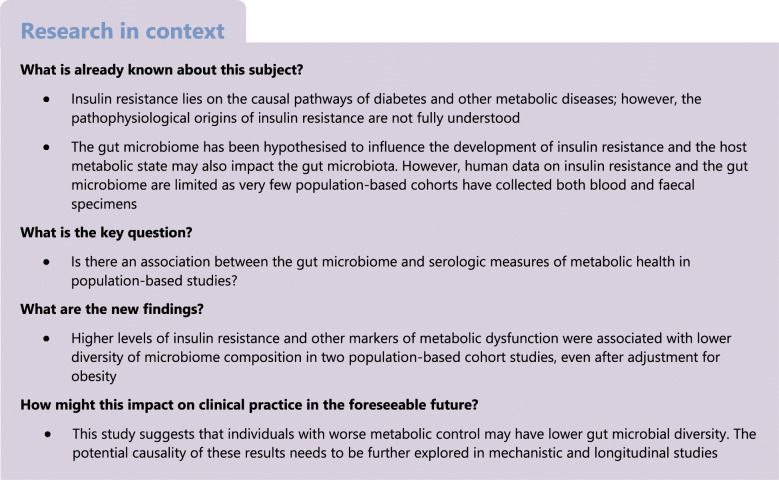



## Introduction

Obesity and type 2 diabetes have reached global epidemic proportions and are recognised as major causes of morbidity and mortality [1, 2]. Insulin resistance is a pathophysiological condition that precedes the development of type 2 diabetes [3]; however, its aetiology is not fully understood. Obesity is a recognised risk factor for insulin resistance but not all insulin-resistant individuals are overweight or obese [4]; indeed, the existence of metabolically healthy obese and metabolically unhealthy normal weight individuals has been described in previous studies [5]. Despite the well-established epidemiological links between insulin resistance, its related variables (i.e., poor glucose control and high levels of inflammation) and chronic diseases including obesity and type 2 diabetes, the potential role of the gut microbiome in the development of insulin resistance and type 2 diabetes is not fully understood.

Previous studies have suggested a link between the gut microbiome and metabolic health [6, 7], and have described differences of microbial composition and functionality in type 2 diabetes patients compared with healthy participants [7, 8]. The gut microbiome is potentially associated with host metabolic health through several pathways including energy extraction, intestinal barrier integrity, metabolism of bile acids and host metabolic and signalling pathways, which are directly or indirectly related to insulin resistance development [9]. For example, circulating levels of branched-chain amino acids (BCAA) have been positively associated with insulin resistance [10] and BCAA levels in insulin-resistant individuals correlate with specific changes in gut microbiome composition and functions, such as enriched potential for BCAA biosynthesis and deprivation of genes encoding for BCAA transport into bacterial cells [11]. However, because of the cross-sectional nature of these studies, it is impossible to conclude whether these differences in microbial composition are a cause or a consequence of metabolic dysfunction.

Despite an important expansion of research on the relationship between the human gut microbiome and metabolic disorders in recent years, human data on the link between the gut microbiota and insulin resistance from population-based studies are lacking. In this study, we investigated the relationship between measures of metabolic health (i.e., insulin resistance, glucose control and inflammation) and gut microbial diversity, independent of BMI, among individuals in the Northern Finland Birth Cohort (NFBC1966) and then sought to replicate these findings in the TwinsUK cohort.

## Methods

### Ethics approval and consent to participate

Informed written consent was obtained from all NFBC1966 participants, and the research protocols were approved by the Ethics Committee of Northern Ostrobotnia Hospital District, Finland. Written informed consent was obtained from all the TwinUK volunteers upon registration and also during their clinical visits, and the research protocols had ethical approval as part of the TwinsUK (EC04/015) study from the Local Research Ethics Committee at the Department of Twin Research and Genetic Epidemiology, King’s College London. The study was approved by the International Agency for Research on Cancer Ethics Committee.

### Northern Finland Birth Cohort

#### Study design and samples

The NFBC1966 was established in 1965 and included 12,055 pregnant women with expected delivery dates between 1 January and 31 December 1966, and subsequently 12,058 children representing 96% of live births in two Finnish provinces, Oulu and Lapland (https://www.oulu.fi/NFBC1966/) [12]. Pregnancies were followed prospectively and children were followed through childhood, adolescence and early adulthood up to 46 years of age. At 31 and 46 years of age, information about health, behaviour, work and social background were collected using self-administered questionnaires and clinical examinations were performed. At 31 and 46 years of age, measures of metabolic health were generated using blood samples. Additionally, at 46 years of age, the participants were asked to collect a stool sample at home using a collection tube without additive that was provided by study investigators. Participants were asked to return the sample to the study facility the same day; if the specimen was taken 1–2 days in advance, the participants were asked to store the stool sample at −20°C. At the study laboratory, the stool samples were first transferred to −20°C freezers and after, to long-term −70°C freezers without any additive within days of collection.

This investigation builds upon a related study conducted by Loftfield et al within the NFBC1966, which aimed to explore associations of BMI history and adult BMI with faecal microbial diversity (*n* = 565) and microbial metabolite levels (*n* = 340) [13]. In the current analysis, participants were selected from the same cohort with faecal microbial diversity data and were included if measures of metabolic health (i.e., insulin [μU/ml], glucose [mmol/l], high-sensitivity C-reactive protein [mg/l], and HbA_1c_ [mmol/mol]) were available at 46 years of age. In total, 506 participants were included in our analytic sample, comprising 187 men (37.0%) and 319 women (63.0%).

At age 46 years, participants underwent clinical examination, during which height and weight were measured, and they completed self-administered questionnaires reporting lifestyle and demographic characteristics. BMI was estimated as measured weight (kg) divided by measured height (m) squared (kg/m^2^). BMI was categorised according to the WHO international classification system: normal weight (18.5–24.9), overweight/pre-obese (25.0–29.9), obese (≥ 30.0). Smoking status was categorised as never, former or current smoker.

#### Laboratory methods

The laboratory methods have previously been described in detail [14]. Metabolic markers were measured for all individuals after an overnight fasting period (12 h). Fasting plasma glucose was analysed by an enzymatic dehydrogenase method (Advia 1800, Siemens Healthcare Diagnostics, Tarrytown, New York, USA). Fasting serum insulin was analysed by a chemiluminometric immunoassay (Advia Centaur XP, Siemens Healthcare Diagnostics, Tarrytown, New York, USA). HOMA for insulin resistance (HOMA-IR) is a commonly used measure of insulin resistance and is calculated as previously described [15]. HOMA-IR values were subdivided into quartiles based on their distribution across all included participants: Q_1_: <1.20, Q_2_: 1.20–1.91, Q_3_: 1.92–3.1, Q_4_: >3.11 for categorical analyses. The concentrations of HbA_1c_ were measured using immunochemical assay methods. High-sensitivity C-reactive protein (CRP) was analysed by an immune-nephelometric assay (BN ProSpec, Siemens Healthcare Diagnostics, Newark, Delaware, USA). HOMA-IR, HbA_1c_ and CRP were log-transformed (natural logarithm) for continuous analyses.

#### DNA extraction, amplification and sequencing

Faecal samples were processed at the University of California, San Diego (La Jolla, California). DNA extraction, PCR amplification and sequencing were completed as described by Loftfield et al [13] and by Vogtmann et al [16], using the universal bacterial primer set 515F/806R [17]. In brief, study samples were randomly ordered and distributed within the batches. For technical reproducibility, replicate faecal samples from three individuals (*n* = 62) were distributed within and across batches. Four quality control samples were also included within each DNA extraction batch: artificial community, chemostat community [18], extraction blank and PCR blank. DNA was extracted with the MO-BIO PowerSoil DNA isolation kit and the V4 region of the 16S rRNA gene was PCR amplified and 2 × 150 bp paired-end sequencing was performed on the MiSeq (Illumina, San Diego, CA). After removing singletons and reads with read errors, the mean coverage was approximately 112,000 reads per sample.

#### Bioinformatics

Processing was performed using QIIME 2 2017.8 [19]. Sequences were demultiplexed, and quality control on forward reads was performed with DADA2 [20]. Paired-end reads were not joined because shorter 16S rRNA gene sequences would be dropped, resulting in systematic bias in community composition. Taxonomy was assigned to amplicon sequence variants (ASVs) using q2-feature-classifier [21] and the Greengenes 13_8 reference database [22]. A phylogenetic tree was built by aligning ASVs with MAFFT [23], filtering highly variable positions via q2-alignment, and applying FastTree [24] to construct an unrooted tree, followed by midpoint rooting using q2-phylogeny midpoint-root. Diversity metrics (i.e., Shannon index, observed sequence variants, binomial deviance dissimilarity, Jaccard, weighted and unweighted UniFrac) were computed using the ‘vegan’ [25] and ‘microbiome’ packages in R (version 3.6.0) at a depth of 10,000 reads per sample to represent the diversity of unique sequences in each sample. As described by Loftfield et al, inspection of quality control data suggested good reproducibility within and across batches [13].

### TwinsUK

#### Study design and samples

The design of the TwinsUK cohort has been described previously [26]. In brief, the TwinsUK cohort is one of the largest adult twin registries comprising over 14,000 volunteers followed over more than two decades. Participants were predominantly female (>80%) and middle-aged (mean age 59). Data were collected during visits to the Department of Twin Research and Genetic Epidemiology, King’s College London, resulting in biochemical, behavioural, dietary and socioeconomic characterisation. In the current study, we analysed data on the gut microbiome and both HOMA-IR and CRP from 1140 female participants. Anthropometric measurements, including height and weight, were measured during each participant’s annual clinic visit, allowing BMI to be calculated. Questionnaires were used to define age (from birthdate) and smoking status.

#### Laboratory methods

Plasma glucose and insulin were measured for all individuals after a 10 h overnight fast. Insulin was quantified by immunoassay (Abbott Laboratories, Maidenhead, UK) and glucose was measured by an Ektachem 700 multichannel analyser using an enzymatic colorimetric slide assay (Johnson and Johnson Clinical Diagnostic Systems, Amersham, UK), as previously described [27]. Highly sensitive CRP was measured by latex-enhanced nephelometry on a Siemens Prospec Nephelometer. HOMA-IR was calculated as described above for NFBC1966. HOMA-IR, HbA_1c_ and CRP were log-transformed (natural logarithm) for continuous analyses.

#### DNA extraction, sequencing and bioinformatics

Faecal samples were collected at home and brought or sent on ice to the clinical research facility where they were stored at −80°C. Samples were then processed to determine gut microbial composition by 16S rRNA gene sequencing, as previously described [28]. Briefly, amplicon PCR was performed on the V4 region of the 16S rRNA gene using the primer pair 515f to 806r with Golay error-correcting barcodes on the reverse primer. The barcoded amplicon pool was purified with the MO-BIO UltraClean PCR cleanup kit and sequenced on the Illumina MiSeq platform. Sequence data were demultiplexed using the QIIME2 2017.8 [19]. ASVs were generated with DADA2 [20]. Diversity metrics (i.e., Shannon index, observed sequence variants, binomial deviance dissimilarity, Jaccard, weighted and unweighted UniFrac) are presented in electronic supplementary material (ESM) Table 1 and were computed using the ‘vegan’ (25) and ‘microbiome’ packages in R (verion 3.6.0).

### Statistical analyses

Descriptive characteristics of the participants were presented by BMI groups. As presented in Table 1, the majority of NFBC1966 participants were female, and 29.2%, 45.9% and 24.9% were normal weight, overweight and obese, respectively. The TwinsUK cohort was entirely female and older than the NFBC1966 cohort (mean age 62.9 and 46.6 years, respectively). Although the NFBC1966 results presented here include both men and women, sensitivity analyses were performed in women only (*n* = 319) with similar results obtained (data not shown). In NFBC1966, 31.9% of current smokers were obese vs 16.5% in TwinsUK.

**Table 1:** Description of the study population stratified by BMI category in NFBC1966 and TwinsUK

	NFBC1966 (*n*=506)			TwinsUK (*n*=1140)		
	BMI, kg/m^2^			BMI, kg/m^2^		
Variable	18.5 to 24.9	25.0 to 29.9	over 30	18.5 to 24.9	25.0 to 29.9	over 30
Total study population	148 (29.2)	232 (45.9)	126 (24.9)	499 (43.8)	411 (36.1)	230 (20.2)
Male	32 (17.1)	108 (57.8)	47 (25.1)	0	0	0
Female	116 (36.4)	124 (38.9)	79 (24.8)	499 (43.7)	411 (36.1)	230 (20.2)
Age, years	46.6 ± 0.6	46.6 ± 0.5	46.7 ± 0.6	62.9 ± 8.9	64.2 ± 8.5	61.8 ± 8.4
Never smoker	98 (33.9)	128 (44.3)	63 (21.8)	300 (43.7)	235 (34.2)	152 (22.1)
Former smoker	29 (23.6)	61 (49.6)	33 (26.8)	152 (45.0)	127 (37.6)	59 (17.5)
Current smoker	21 (22.3)	43 (45.8)	30 (31.9)	47 (40.9)	49 (42.6)	19 (16.5)
HOMA-IR	1.32 ± 0.7	2.26 ± 1.3	4.61 ± 4.4	0.84 ± 1.32	1.12 ± 1.46	1.56 ± 1.37
CRP, mg/l	1.08 ± 1.7	1.45 ± 2.7	2.95 ± 6.6	1.88 ± 2.95	2.89 ± 6.29	5.17 ± 8.41
HbA_1c_, mmol/mol	33.20 ± 2.0	34.02 ± 4.2	35.03 ± 3.6	*–*	*–*	*–*
HbA_1c_, %	5.2	5.3	5.4	–	–	–

Data are shown as *n* (%) or mean ± SD

Spearman correlations were calculated to estimate correlations between the different measures of metabolic health and BMI. In NFBC1966, BMI was strongly correlated with HOMA-IR (*R* = 0.64; 95% CI 0.58, 0.68) and more moderately correlated with CRP (*R* = 0.37; 95% CI 0.29, 0.44) and HbA_1c_ (*R* = 0.23; 95% CI 0.14, 0.31) (ESM Fig. 1). In TwinsUK, BMI was moderately correlated with HOMA-IR (*R* = 0.43; 95% CI 0.38, 0.47) and moderately correlated with CRP (*R* = 0.37; 95% CI 0.31, 0.41). Prior to conducting multivariable analyses, variance inflation factors (VIF) were calculated to evaluate potential multicollinearity between alpha diversity metrics, BMI and HOMA; moderate VIF were found with these two covariates (VIF BMI 1.37, VIF HOMA-IR 1.43), suggesting that these predictors are not correlated with other variables. To estimate the association of the measures of metabolic health as categorical and continuous variables with the alpha diversity metrics, general linear models with a quasi-Poisson distribution were used. The estimated value in Table 2 signifies how much the mean of the dependent variable (measures of microbiome diversity) changes given a one-unit shift in the independent variable (markers of metabolic health) while holding other variables in the model constant. In the NFBC1966, no visual clustering was observed by measures of metabolic health using the first three principal coordinate analysis vectors from four beta diversity matrices (results not shown). For the association between microbial community profiles using beta diversity metrics and the measures of metabolic health, MiRKAT tests were performed [29]. To identify the taxa associated with HOMA-IR, CRP and HbA_1c_, after grouping ASVs at the genus level, a generalised additive model for location scale and shape (GAMLSS) fitted with the zero-inflated beta distribution was computed using the ‘gamlss’ package in R [30]. Likelihood-ratio tests between models including adjustment factors only and models including the measures of metabolic health and adjustment factors were performed. *P* values <0.05 were considered as indicators of an association between metabolic health and distribution of the taxonomic component. The *p* values were then adjusted using a Benjamini–Hochberg false discovery rate (FDR) <0.05. All statistical tests described above were adjusted for BMI, sex and smoking status in the NFBC1966 cohort and for BMI, age and smoking status in the TwinsUK cohort. Other possible confounders (i.e., education, alcohol, fruit, vegetables, cereals, fish, red and processed meat, poultry, dairy and physical activity) were considered but not selected in the final models, based on a bidirectional stepwise selection.

**Table 2:** Associations of metabolic variables (continuous and categorical) with measures of alpha diversity in NFBC1966 and TwinsUK

	NFBC1966 (n=506)^a^				TwinsUK (n=1140)^b^			
	Shannon’s diversity		Observed ASVs		Shannon’s diversity		Observed ASVs	
Variable	Estimate	*p* value	Estimate	*p* value	Estimate	*p* value	Estimate	*p* value
HOMA-IR								
Continuous	−0.047	0.001	−0.072	0.002	−0.062	<0.001	−0.106	0.001
Quartile 1	Ref	Ref	Ref	Ref	Ref	Ref	Ref	Ref
Quartile 2	−0.022	0.282	−0.033	0.304	−0.021	0.051	−0.019	0.413
Quartile 3	−0.019	0.379	−0.055	0.118	−0.027	0.017	−0.041	0.083
Quartile 4	−0.078	0.003	−0.111	0.008	−0.055	<0.001	−0.093	<0.001
CRP								
Continuous	−0.016	0.085	−0.032	0.025	−0.018	0.031	−0.041	0.019
Quartile 1	Ref	Ref	Ref	Ref	Ref	Ref	Ref	Ref
Quartile 2	−0.031	0.154	−0.007	0.843	−0.007	0.523	−0.01	0.657
Quartile 3	−0.023	0.303	−0.028	0.415	−0.006	0.599	−0.009	0.688
Quartile 4	−0.027	0.263	−0.049	0.195	−0.028	0.016	−0.063	0.01
HbA_1c_								
Continuous	−0.042	0.003	−0.113	<0.001	*–*	*–*	*–*	*–*
Quartile 1	Ref	Ref	Ref	Ref	*–*	*–*	*–*	*–*
Quartile 2	0.039	0.099	0.008	0.822	*–*	*–*	*–*	*–*
Quartile 3	0.005	0.832	−0.038	0.256	*–*	*–*	*–*	*–*
Quartile 4	−0.021	0.347	−0.093	0.007	*–*	*–*	*–*	*–*

^a^Adjusted for BMI, sex and smoking status

^b^Adjusted for BMI, age and smoking status

## Results

### Alpha diversity

Overall, alpha diversity declined with increasing quartiles of HOMA-IR in the NFBC1966 (Table 2). For example, the mean number of ASVs was lower in the 4th quartile of HOMA-IR (66.6, 95% CI 62.9, 70.4) compared with the 1st quartile (74.4, 95% CI 70.7, 78.3) (ESM Table 2). Similar results were found in the TwinsUK cohort where Shannon’s diversity was lower in the 4th quartile of HOMA-IR (3.72, 95% CI 3.65, 3.79) than the 1st quartile (3.91, 95% CI 3.84, 3.98) (ESM Table 2). In NFBC1966, the number of observed ASVs was inversely associated with HOMA-IR (estimate per unit increase = −0.072, *p* = 0.002), CRP (estimate per unit increase = −0.032, *p* = 0.025) and HbA_1c_ levels (estimate per unit increase = −0.113, *p* < 0.001) in continuous models. Shannon’s diversity was also inversely associated with HOMA-IR (estimate per unit increase = −0.047, *p* = 0.001) and HbA_1c_ levels (estimate per unit increase = −0.042, *p* = 0.003) in NFBC1966. In TwinsUK, Shannon’s diversity was inversely associated with HOMA-IR (estimate per unit increase = −0.062, *p* < 0.001) and CRP (estimate per unit increase = −0.018, *p* = 0.031). The number of observed ASVs was also inversely associated with HOMA-IR (estimate per unit increase = −0.106, *p* = 0.001) and CRP (estimate per unit increase = −0.041, *p* = 0.019). In the NFBC1966, the association between BMI and the number of observed ASVs was no longer significant after adjustment for HOMA-IR (*p* value without HOMA-IR: 0.001, *p* value in model including HOMA-IR: 0.489) and CRP (*p* value in model including CRP: 0.082) (ESM Table 3). However, the association between BMI and the number of observed ASVs remained significant in multivariable models including HbA_1c_. In the TwinsUK cohort, BMI remained strongly inversely associated with alpha diversity following adjustments for HOMA-IR or CRP (ESM Table 3).

### Beta diversity

HOMA-IR, CRP and HbA_1c_ were associated with all community composition measures except for weighted UniFrac and binomial deviance dissimilarity for CRP in the NFBC1966 (Table 3). Similar results were found for HOMA-IR in the TwinsUK cohort. However, CRP was only associated with Jaccard (*p =* 0.043) in TwinsUK. BMI was associated with all community composition measures except for weighted UniFrac in the NFBC1966 cohort and with all beta diversity measures in TwinsUK (ESM Table 4). However, the associations between BMI and microbial composition for binomial and unweighted UniFrac metrics were no longer significant after adjustment for HOMA-IR in NFBC1966 (ESM Table 4). BMI remained strongly associated with four beta diversity matrices, regardless of the inclusion of HOMA-IR or CRP in the model in the TwinsUK cohort.

**Table 3:** Association of metabolic variables with community composition using measures of beta diversity in NFBC1966 and TwinsUK

	NFBC1966 (n=506)^a^				TwinsUK (n=1140)^b^			
Variable	Binomial *p* value	Jaccard *p* value	Weighted UniFrac *p* value	Unweighted UniFrac *p* value	Binomial *p* value	Jaccard *p* value	Weighted UniFrac *p* value	Unweighted UniFrac *p* value
HOMA-IR	<0.001	<0.001	0.563	0.004	<0.001	<0.001	0.072	<0.001
CRP	0.112	0.007	0.384	0.047	0.345	0.043	0.241	0.099
HbA_1c_	<0.001	<0.001	0.112	<0.001	*–*	*–*	*–*	*–*

^a^Adjusted for BMI, sex and smoking status

^b^Adjusted for BMI, age and smoking status

### Relative abundance at the genus level

In the NFBC1966 cohort, 16 individual genera met FDR-adjusted significance level (<0.05) for their associations with HOMA-IR, ten with CRP and nine with HbA_1c_. Nineteen individual genera met FDR-adjusted significance (<0.05) with HOMA-IR and eight with CRP in the TwinsUK cohort. Higher values of HOMA-IR were strongly associated with higher mean relative abundances of members of the Peptococcaceae (estimate per unit increase = 11.62, *p* < 0.001), Bifidobacteriaceae (genus *Gardnerella*) (estimate per unit increase = 2.64, *p* < 0.001), Veillonellaceae, Lachnospiraceae (genus *Blautia*) and Prevotellaceae (genus *Paraprevotella*) families and lower mean relative abundances of members from the Peptostreptococcaceae (estimate per unit increase = −0.52, *p* < 0.001), Peptococcaceae (estimate per unit increase = −0.23, *p* < 0.001), and Prevotellaceae (genus *Prevotella*) families (ESM Table 5) in the NFBC1966 cohort. Higher values of HOMA-IR were also associated with higher mean relative abundances of members from Lachnospiraceae (genus *Blautia*) and Prevotellaceae (genus *UCG-003*) and lower mean relative abundances of *Prevotella* in TwinsUK (ESM Table 6). Higher CRP was associated with lower mean relative abundances of members from the Paraprevotellaceae, Peptococcaceae, Veillonellaceae and Peptostreptococcaceae families and higher mean relative abundance of one genus, *Peptococcus* in NFBC1966 (ESM Table 5). Higher levels of CRP were associated with higher mean relative abundances of members from the Enterobacteriaceae, Elusimicrobiaceae, Erysipelotrichaceae and Prevotellaceae families and lower mean relative abundance of *Dysgonomonas* and NK4A214 group (family Ruminococcaceae) in TwinsUK (ESM Table 6). Higher levels of HbA_1c_ were associated with lower mean relative abundances of members from the Bifidobacteriaceae, Peptococcaceae, Veillonellaceae and Peptostreptococcaceae families and higher mean relative abundance of one genus, *Oxalobacter*, in the NFBC1966 cohort (ESM Table 5). The metabolic biomarkers were not associated with the presence/absence of individual genera except for the genus *Gardneralla* and HbA_1c_ (estimate per unit increase = 3.87, *p* = 0.013) in NFBC1966. Higher values of HOMA-IR were associated with higher presence of members of the Lachnospiraceae family and *Veillonella* in TwinsUK.

## Discussion

In this analysis of gut microbiome profiles and metabolic variables in two population-based cohorts, higher levels of HOMA-IR, CRP and HbA_1c_ were associated with lower diversity of the gut microbiome, even after adjustment for BMI and other factors. In addition, HOMA-IR, CRP and HbA_1c_ were associated with differences in microbial community between individuals as indicated by associations with three beta diversity measures, but were not associated with weighted UniFrac, suggesting that the metabolic biomarkers were associated with differences in the presence or absence of bacteria in different communities but not with differences in abundance.

The unweighted UniFrac measure allows the detection of differences in the presence or absence of lineages of bacteria in different communities. On the other hand, the weighted UniFrac measure helps to detect differences in abundance even when the overall groups of organisms that are present in each sample remain the same [31]. In weighted UniFrac, low-abundance taxa have a much lower weight than in unweighted UniFrac and so will have a lower impact on the total distance reported by the metric. Further, HOMA-IR, CRP and HbA_1c_ were mainly associated with the relative abundance of specific genera, and less with their presence or absence, indicating that the associations between the metabolic biomarkers and microbiome diversity captured by beta diversity measures were potentially driven by a consortium of bacteria present at low relative abundance. In addition, although only observed in the NFBC1966 cohort, in an exploratory analysis we found that the association of BMI and microbiome diversity was attenuated when adjusting the model for HOMA-IR, supporting that some degree of the BMI–microbiome relation might be explained by the association of insulin resistance with microbial diversity.

Our data support the hypothesis that greater gut microbiome diversity is associated with better insulin sensitivity [6]. A clinical trial studying the effect of faecal transplant from lean donors to men diagnosed with the metabolic syndrome found that insulin sensitivity had improved and gut bacterial diversity had increased 6 weeks post transplantation [32]. These results corroborate other studies identifying gut microbial alterations and attenuation of the metabolic syndrome after various weight-loss interventions [33, 34]. As described previously, the gut microbiome can trigger inflammatory processes associated with obesity and insulin resistance by stimulating immune cells through lipopolysaccharides derived from bacterial membranes [35, 36]. Furthermore, microbial-derived short-chain fatty acids, including butyrate [37], can enhance insulin sensitivity [38] and suppress insulin-mediated fat accumulation [39]. CRP is a marker of chronic low-grade systemic inflammation associated with obesity and insulin resistance [40]. Previous studies have found a similar relationship between systematic inflammation, through analysis of high-sensitivity CRP plasma levels and reduced bacterial diversity, supporting our findings [41]. Our results suggest that higher levels of HbA_1c_ were also associated with lower bacterial diversity. Existing literature on the association between HbA_1c_ level and gut microbiome composition are inconsistent. Some studies reported evidence of an association between HbA_1c_ level and bacterial group counts [42] while others did not find any relationship [43]. These findings could be explained by the fact that elevated insulin levels or HOMA-IR appeared to identify certain traits of the metabolic syndrome, especially abdominal obesity, earlier than that seen in changes to both HbA_1c_ and measures of glucose [44].

Consistent with other studies, BMI was inversely associated with gut microbiome diversity in this study [13]. However, following adjustment for HOMA-IR and CRP, the associations between BMI and some measures of alpha- and beta diversity were no longer detected, suggesting that the observed relationship between BMI and gut microbiome diversity might be indirect and influenced by measures of insulin resistance and inflammation. This hypothesis needs to be further explored in other cohorts as results from TwinsUK suggested that associations between BMI and gut microbial diversity were attenuated but remained significant.

The relative abundance of individual genera was associated with HOMA-IR (16 and 19 genera in NFBC1966 and TwinsUK, respectively), with CRP (ten and eight genera in NFBC1966 and TwinsUK, respectively) and with HbA_1c_ (nine genera in NFBC1966). Overall, the GAMLSS models showed that metabolic biomarkers were more associated with the relative abundance of the taxa rather than their presence or absence. At the genus level, *Blautia* was positively and *Prevotella* inversely associated with HOMA-IR in both cohorts. In previous studies, *Blautia* has been reported to be related to disturbances in glucose metabolism and type 2 diabetes [45–48]. At the mechanistic level, exposure of human tissue to *Blautia* species has been shown to induce an inflammatory response greater than that induced by lipopolysaccharide stimulation [49]. In addition, in a rat diabetes model, exposure to *Blautia* was positively correlated with inflammatory indicators including, IL-1β, TNF-α, IL-6 and lipopolysaccharides [50]. These results suggest that *Blautia* might be related to metabolic health through its effects on specific inflammatory pathways. Lower abundance of *Prevotella* in diabetic patients was previously observed, consistent with our data on insulin resistance and metabolic health [51–53]. In addition, *Prevotella* was shown to be positively associated with dietary fibre-induced improvement in glucose metabolism by potentially playing a role in the fermentation of complex polysaccharides and in the storage of glycogen [54]. In contrast, increased abundance of *Prevotella* strains were associated with insulin resistance in a non-diabetic cohort [11] with obesity [55, 56] and with low-grade inflammation in the gut [57]. However, it was speculated that these effects may not be causally linked to the presence of *Prevotella* and that other members of the *Prevotella*-dominated microbiome may have the propensity to promote inflammation [58] Overall, these findings indicate that *Blautia* and *Prevotella* may induce biological changes particularly in inflammatory pathways that may explain their association with metabolic health in our study. Further experimental studies are needed to identify mechanisms by which these bacteria may be related to host metabolic health.

A limitation of our study was the cross-sectional nature of the analyses. Repeated, prospectively collected samples are now needed to identify potential causal relationships between gut microbiome and markers of metabolic health and to assess their respective association with risk of diseases related to metabolic dysfunction such as type 2 diabetes or certain cancers. Other limitations were that TwinsUK only included female participants, although sensitivity analyses restricted to women in the NFBC1966 showed similar results. Further, data on HbA_1c_ were not available in TwinsUK to validate the NFBC1966 results. We also note differences between the studied NFBC1966 and TwinsUK populations in terms of age, obesity prevalence and metabolic variables. However, being able to observe consistent associations between gut microbiome composition and measures of metabolic health in two different populations and independently of other potential factors such as age and obesity may indicate the results are generalisable across different populations. An additional limitation is that the observed associations of microbiome diversity measures with metabolic health biomarkers in both cohorts may have also been inflated or attenuated by unmeasured or poorly measured confounders, such as dietary and fibre intake and the use of medication. Finally, we recognise the limited taxonomic resolution and accuracy of the 16S rRNA gene sequencing methodology, preventing us from adequately performing species-level associative analysis and resulting in a limited overlap between the two cohorts when focusing on associations with individual genera. To unambiguously discriminate low-abundance taxa from noise and further replication and extension (e.g., metagenomics) of results in other population-based studies is warranted.

Our study suggests that general population cohorts are valuable in identifying potential associations between microbial features and measures of metabolic health. However, the use of cross-sectional data does not allow us to causally interpret these associations, since interactions between metabolic biomarkers and the gut microbiome are complex and dynamic and can be strongly affected by behavioural changes. Therefore, it is now critical for future studies to collect longitudinal data of both lifestyle exposures and the microbiome to help understand the dynamic relationship between the gut microbiome and host metabolism. Integration of microbiome data paired with faecal metabolomics data may also provide a more complete picture of the metabolic microbial mechanisms that contribute to metabolic balance between the host and the gut microbiome.

In conclusion, markers of insulin resistance, poor control of blood glucose levels and systemic inflammation were associated with lower gut microbiome diversity and distinct microbial community structures in both the NFBC1966 and TwinsUK cohorts, even after adjustment for BMI and other variables. These results from two distinct population-based cohorts provide evidence that individuals with worse metabolic control have lower gut microbial diversity. It is, however, impossible to conclude if these differences in microbial composition and taxa associations are a cause or a consequence of metabolic dysfunction. Thus, large-scale, prospective studies with collection of faecal samples and longitudinal data on lifestyle and metabolic biomarkers at several time-points are now needed to validate and extend these observations.

## Supplementary information

ESM (PDF 196 kb)

## Data Availability

The datasets used and/or statistical analysis code for the current study are available from the authors (Marc Gunter, gunterm@iarc.fr) on reasonable request.

## Abbreviations

ASV: Amplicon sequence variants
BCAA: Branched-chain amino acids
CRP: C-reactive protein
FDR: False discovery rate
GAMLSS: Generalised additive models for location scale and shape
MiRKAT: Microbiome Regression-based Kernel Association Test
NFBC1966: Northern Finland Birth Cohort
VIF: Variance inflation factors
