# Supplementary material for: Markers of metabolic health and gut microbiome diversity: findings from two population-based cohort studies
Source: Diabetologia. 2021 Jun 10;64(8):1749–59. doi: 10.1007/s00125-021-05464-w (PMC8245388; doi:10.1007/s00125-021-05464-w)

ESM Table 1 – Description of the ecological diversity measures used to describe microbiome diversity in the NFBC1966 and TwinsUK

Diversity indices	Description	References
**Shannon diversity index**	Based on species richness and species evenness: more weight on species richness	[1]
**Observed ASVs**	Based on species richness: number of species present in a community	[2]
**Binomial deviance dissimilarity**	Based on abundance: differences in microbial abundances between two samples	[3]
**Jaccard index**	Based on presence or absence of species: differences in microbial composition between two samples	[4]
**Unweighted UniFrac**	Based on sequence distances: fraction of branch length shared/unshared between two samples - based on the presence or absence of observed organisms	[5]
**Weighted UniFrac**	Based on the relative abundance of observed organisms to calculate shared/unshared branch lengths between two samples	[5]

1. Lemos LN, Fulthorpe RR, Triplett EW, Roesch LFW (2011) Rethinking microbial diversity analysis in the high throughput sequencing era. J Microbiol Methods 86(1):42–51. https://doi.org/10.1016/j.mimet.2011.03.014

2. Hughes JB, Hellmann JJ, Ricketts TH, Bohannan BJM (2001) Counting the Uncountable: Statistical Approaches to Estimating Microbial Diversity. Appl Environ Microbiol 67(10):4399–4406. https://doi.org/10.1128/AEM.67.10.4399-4406.2001

3. Anderson MJ, Millar RB (2004) Spatial variation and effects of habitat on temperate reef fish assemblages in northeastern New Zealand. Journal of Experimental Marine Biology and Ecology 305(2):191–221. https://doi.org/10.1016/j.jembe.2003.12.011

4. Levandowsky M, Winter D (1971) Distance between Sets. Nature 234(5323):34–35. https://doi.org/10.1038/234034a0

5. Lozupone C, Lladser ME, Knights D, Stombaugh J, Knight R (2011) UniFrac: an effective distance metric for microbial community comparison. ISME J 5(2):169–172. https://doi.org/10.1038/ismej.2010.133

ESM Table 2 - Association of metabolic parameters (categorical) with measures of alpha-diversity in NFBC1966 and TwinsUK

	NFBC1966 (n=506)[a]		TwinsUK (n=1140)[b]	
	Shannon's diversity	Observed ASVs	Shannon's diversity	Observed ASVs
	Least-squares mean (95% CI)	Least-squares mean (95% CI)	Least-squares mean (95% CI)	Least-squares mean (95% CI)
**HOMA-IR**				
Quartile 1	2.80 (2.71; 2.89)	74.4 (70.7; 78.3)	3.91 (3.84;3.98)	214 (206; 223)
Quartile 2	2.74 (2.65; 2.83)	71.9 (68.4; 75.6)	3.83 (3.77; 3.98)	211 (203; 220)
Quartile 3	2.75 (2.67; 2.83)	70.4 (67.1; 73.9)	3.82 (3.75; 3.89)	207 (199; 216)
Quartile 4	2.59 (2.50; 2.68)	66.6 (62.9; 70.4)	3.72 (3.65; 3.79)	198 (190; 206)
**CRP**				
Quartile 1	2.76 (2.67; 2.85)	71.6 (68.1; 75.3)	3.85 (3.78; 3.93)	211 (203; 220)
Quartile 2	2.67 (2.59; 2.76)	71.1 (67.7; 74.7)	3.83 (3.76; 3.90)	210 (202; 218)
Quartile 3	2.70 (2.62; 2.78)	69.6 (66.2; 73.1)	3.84 (3.77; 3.91)	210 (202; 219)
Quartile 4	2.69 (2.60; 2.78)	68.2 (64.6; 72.0)	3.77 (3.70; 3.84)	201 (193; 209)
**HbA_{1c}**				
Quartile 1	2.71 (2.62; 2.81)	73.6 (69.8; 77.6)	-	-
Quartile 2	2.82 (2.72; 2.92)	74.2 (70.2; 78.4)	-	-
Quartile 3	2.72 (2.65; 2.80)	70.9 (67.9; 73.9)	-	-
Quartile 4	2.66 (2.58; 2.73)	67.0 (64.1; 70.1)	-	-

[a]Adjusted for BMI, gender and smoking status
[b]Adjusted for BMI, age and smoking status

ESM Table 3 – Association of measures of alpha-diversity with BMI before and after adjustment for metabolic markers in NFBC1966 and the TwinsUK

Model adjusted for	NFBC1966 (n=506)[α]				TwinsUK (n=1140)[β]			
	Shannon's diversity		Observed ASVs		Shannon's diversity		Observed ASVs	
	Estimate	P-value[α]	Estimate	P-value[α]	Estimate	P-value[β]	Estimate	P-value[β]
Standard adjustment	-0.002	0.258	-0.008	0.001	-0.004	<0.001	-0.009	<0.001
HOMA-IR	0.002	0.253	-0.002	0.489	-0.002	0.006	-0.007	<0.001
CRP	0.000	0.972	-0.005	0.082	-0.003	<0.001	-0.008	<0.001
HbA_{1c}	-0.001	0.564	-0.006	0.015	-	-	-	-

[α]Adjusted for BMI, gender and smoking status

[β]Adjusted for BMI, age and smoking status

ESM Table 4 – Association of BMI, before and after adjustment for metabolic markers, with community composition using measures of beta-diversity in NFBC1966 and TwinsUK

Model adjusted for	NFBC1966 (n=506)[α]				TwinsUK (n=1140)[β]			
	Binomial P-value	Jaccard P-value	Weighted Unifrac P-value	Unweighted Unifrac P-value	Binomial P-value	Jaccard P-value	Weighted Unifrac P-value	Unweighted Unifrac P-value
Standard adjustment	0.004	<0.001	0.113	0.004	<0.001	<0.001	<0.001	<0.001
HOMA-IR	0.099	0.004	0.241	0.258	<0.001	<0.001	0.001	<0.001
CRP	0.015	<0.001	0.011	0.004	<0.001	<0.001	<0.001	<0.001
HbA_{1c}	0.015	<0.001	0.09	0.037	-	-	-	-

[α]Adjusted for gender and smoking status

[β]Adjusted for age and smoking status

ESM Table 5a - Association of HOMA-IR with genera in NFBC1966

		Mu (mean parameter)		Nu (probability at zero)	
**Family**	**Genus**	**Estimate**	**Adj. p-value**	**Estimate**	**Adj. p-value**
**Desulfovibrionaceae**	**Desulfovibrio**	-0.0223	<0.0001	-0.0833	0.5113
**Veillonellaceae**	**Megamonas**	1.3963	<0.0001	0.0402	0.9982
**Peptostreptococcaceae**	**Unknown**	-0.5189	<0.0001	-0.0112	0.9563
**Peptococcaceae**	**rc4-4**	11.6284	<0.0001	0.8720	0.3907
**Prevotellaceae**	**Paraprevotella**	0.0494	<0.0001	-0.1222	0.1463
**Peptococcaceae**	**Peptococcus**	-0.2263	<0.0001	0.1305	0.7036
**Mogibacteriaceae**	**Unknown**	-0.0570	<0.0001	0.0988	0.0943
**Bifidobacteriaceae**	**Gardnerella**	2.6445	<0.0001	-0.0415	0.9995
**Veillonellaceae**	**Mitsuokella**	0.1647	<0.0001	-0.1227	0.1025
**Prevotellaceae**	**Prevotella** (#)	-0.1753	0.0006	-0.0214	0.8095
**Ruminococcaceae**	**Oscillospira**	-0.0779	0.0056	-0.0953	0.7753
**Lachnospiraceae**	**Blautia** (#)	0.0633	0.0121	0.0475	1.0000
**Ruminococcaceae**	**Butyricicoccus**	0.0308	0.8515	-0.1017	0.3426
**Odoribacteraceae**	**Odoribacter**	-0.0069	0.8671	-0.1058	0.1664
**Corynebacteriaceae**	**Corynebacterium**	0.0409	0.9995	0.0415	0.9995
**Actinomycetaceae**	**Arcanobacterium**	1.0614	0.9997	-3.9031	0.9997

Adjusted for BMI, gender and smoking status; the first component of the GAMLSS model is linked with the nu parameter that models the presence or absence of specific genera, while the second component is indexed by the mu parameter that models the relative abundance of these genera; (#) also observed in TwinsUK

ESM Table 5b - Association of CRP with genera in NFBC1966

		Mu (mean parameter)		Nu (probability at zero)	
**Family**	**Genus**	**Estimate**	**Adj. p-value**	**Estimate**	**Adj. p-value**
**Veillonellaceae**	**Megamonas**	-0.2106	<0.0001	-0.0192	0.9747
**Peptococcaceae**	**rc4-4**	-0.8197	<0.0001	0.4157	0.9999
**Prevotellaceae**	**Paraprevotella**	-0.1915	<0.0001	-0.0188	0.9970
**Prevotellaceae**	**Unknown**	-1.2657	<0.0001	0.1757	0.3926
**Peptostreptococcaceae**	**Unknown**	-0.6372	<0.0001	0.1023	0.7983
**Peptococcaceae**	**Peptococcus**	0.2825	<0.0001	0.0449	0.8639
**Alcal**	**Sutterella**	-0.4148	0.0001	0.0514	0.7581
**Odoribacteraceae**	**Odoribacter**	-0.1705	0.2070	0.4615	0.2738
**Ruminococcaceae**	**Butyricicoccus**	7.6385	0.2334	0.2016	0.8018
**Actinomycetaceae**	**Arcanobacterium**	0.0864	1.0000	-0.4791	1.0000

Adjusted for BMI, gender and smoking status

ESM Table 5c - Association of HbA_{1c} with genera in NFBC1966

		Mu (mean parameter)		Nu (probability at zero)	
**Family**	**Genus**	**Estimate**	**Adj. p-value**	**Estimate**	**Adj. p-value**
**Peptostreptococcaceae**	**Unknown**	-1.9110	<0.0001	-0.0227	0.9729
**Peptococcaceae**	**rc4-4**	-0.7623	<0.0001	1.7686	0.5390
**Peptococcaceae**	**Peptococcus**	-2.4020	<0.0001	0.7325	0.4174
**Veillonellaceae**	**Mitsuokella**	-2.0149	<0.0001	-0.0237	0.9547
**Bifidobacteriaceae**	**Gardnerella**	-34.2932	<0.0001	3.8717	0.0132
**Oxalobacteraceae**	**Oxalobacter**	1.4136	0.0014	0.0874	0.9224
**Ruminococcaceae**	**Butyricicoccus**	0.8421	0.3082	-0.4044	0.4060
**Desulfovibrionaceae**	**Desulfovibrio**	-0.5572	0.3199	0.9462	0.3199
**Odoribacteraceae**	**Odoribacter**	-0.2741	0.5934	-0.2817	0.4788

Adjusted for BMI, gender and smoking status

ESM Table 6a - Association of HOMA-IR with genera in TwinsUK

Family	Genus	Mu (mean parameter)		Nu (probability at zero)	
		Estimate	Adj. p-value	Estimate	Adj. p-value
**Lachnospiraceae**	**Blautia** (#)	0.386	<0.0001	0.000	1.000
**Enterobacteriaceae**	**Raoultella**	-3.650	<0.0001	-1.220	0.257
**Enterobacteriaceae**	**Shigella**	0.990	<0.0001	-0.090	0.839
**Enterobacteriaceae**	**Pluralibacter**	18.800	<0.0001	4.770	0.399
**Elusimicrobiaceae**	**Elusimicrobium**	-26.600	<0.0001	-0.741	0.999
**Coriobacteriaceae**	**Collinsella**	0.585	0.012	-0.065	0.780
**Ruminococcaceae**	**UCG-014**	0.140	0.380	-2.270	0.026
**VeillonellaceaeVeillonella**	**Veillonella**	0.423	0.160	1.980	0.003
**Prevotellaceae**	**UCG-003**	0.524	<0.0001	0.983	0.902
**Prevotellaceae**	**Prevotella** (#)	-1.420	0.001	-0.277	0.516
**Streptococcaceae**	**Streptococcus**	0.545	0.008	-0.181	0.773
**Peptostreptococcaceae**	**Terrisporobacter**	0.660	0.007	0.311	0.257
**Lactobacillaceae**	**Lactobacillus**	1.400	0.032	-0.042	0.862
**Lachnospiraceae**	**Lachnospira**	-0.270	0.086	1.700	<0.0001
**Lachnospiraceae**	**GCA-900066575**	0.317	0.057	0.684	0.012
**Ruminococcaceae**	**Ruminococcus**	-0.561	<0.0001	0.611	0.160
**Dysgonomonadaceae**	**Dysgonomonas**	1.470	<0.0001	-0.118	1.000
**Ruminococcaceae**	**Faecalibacterium**	-0.228	0.103	-0.617	0.637
**Ruminococcaceae**	**Intestinimonas**	0.281	0.055	0.593	0.055

Adjusted for BMI, age and smoking status; (#) also observed in the NFBC1966

ESM Table 6b - Association of CRP with genera in TwinsUK

Family	Genus	Mu (mean parameter)		Nu (probability at zero)	
		Estimate	Adj. p-value	Estimate	Adj. p-value
**Enterobacteriaceae**	**Rosenbergiella**	1.530	<0.0001	-0.065	1.000
**Enterobacteriaceae**	**Shigella**	0.438	0.001	-0.140	0.673
**Elusimicrobiaceae**	**Elusimicrobium**	4.630	<0.0001	-1.220	0.310
**Erysipelotrichaceae**	**UCG-006**	1.170	<0.0001	1.740	0.107
**Prevotellaceae**	**UCG-003**	0.459	<0.0001	1.840	0.174
**Dysgonomonadaceae**	**Dysgonomonas**	-0.284	<0.0001	-1.070	0.412
**Ruminococcaceae**	**NK4A214**	-0.291	<0.0001	-0.075	0.743
**Lachnospiraceae**	**UCG-003**	-0.375	0.144	0.706	0.001

Adjusted for BMI, age and smoking status

ESM Figure 1 - Heatmap of Spearman correlation matrix of the measures of metabolic health and BMI in the NFBC1966

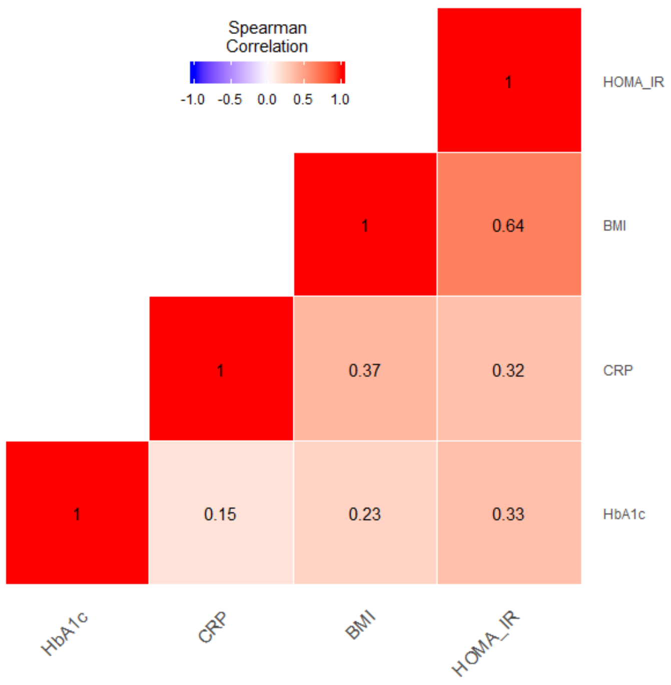

Supplement: Supplementary file 1 — (PDF 196 kb) [file 125_2021_5464_MOESM1_ESM.pdf]
